# Supplementary material for: Human and climatic drivers affect spatial fishing patterns in a multiple-use marine protected area: The Galapagos Marine Reserve
Source: PLoS One. 2020 Jan 23;15(1):e0228094. doi: 10.1371/journal.pone.0228094 (PMC6977758; doi:10.1371/journal.pone.0228094)
Supplement: S1 Table — (DOCX) [file pone.0228094.s001.docx]

**S1 Table. Summary of the fishery monitoring data gathered for the spiny lobster fishery at the three main ports of the Galapagos Marine Reserve from 1997 to 2011.** Total fishing effort was calculated by dividing the annual total catch (in tail kg) by the annual average catch per unit effort (CPUE), with the latter expressed in tail kg diver^-1^ hour ^-1^. Then, we estimated the number and percentage of active small-vessels and effective fishing effort sampled through interviews and observers onboard per fishing season. NA= Not available. Source: Participatory Programme of Fisheries Monitoring and Research (PIMPP, in Spanish), Moreno et al. [40]; Reyes and Ramírez [29].

| **Fishing season** | **Small vessels** | | | **Catch**  **(tail t)** | **CPUE**  **(tail kg diver^-1^ hour^-1^)** | **Fishing effort**  **(diver hours)** | | |
| --- | --- | --- | --- | --- | --- | --- | --- | --- |
|  | **Active** | **Sampled by interviews**  **(% of total)** | **Sampled by observers**  **(% of total)** |  |  | **Total** | **Sampled by interviews**  **(% of total)** | **Sampled by observers**  **(% of total)** |
| 1997 | 147 | 83 (56) | 0 | 65.2 | 1.7 | 38349 | 4064 (11) | 0 |
| 1998 | 147^1^ | 78 (53) | 0 | 30.8 | 1.3 | 24150 | 3833 (16) | 0 |
| 1999 | 194 | 140 (72) | 0 | 52.8 | 1.8 | 29589 | 7963 (27) | 0 |
| 2000 | 286 | 244 (85) | 18 (6) | 82.8 | 2.1 | 38952 | 16587 (43) | 1283 (3) |
| 2001 | 287 | 236 (82) | 53 (18) | 64.5 | 1.7 | 38156 | 14721 (39) | 2352 (6) |
| 2002 | 276 | 196 (71) | 49 (18) | 50.1 | 1.3 | 38454 | 10521 (27) | 2483 (6) |
| 2003 | 228 | 184 (81) | 32 (14) | 45.9 | 1.2 | 37680 | 10156 (27) | 1398 (4) |
| 2004 | 280 | 81 (29) | 36 (13) | 25.7 | 1.1 | 22533 | 2141 (10) | 1260 (6) |
| 2005 | 245 | 30 (12) | 63 (26) | 34.3 | 1.2 | 28105 | 283 (1) | 1892 (7) |
| 2006 | 177 | 45 (25) | 62 (35) | 29.6 | 1.4 | 21833 | 2801 (13) | 2221 (10) |
| 2007 | NA | 0 | 0 | 30.2 | NA | NA | 0 | 0 |
| 2008 | 150 | 102 (68) | 0 | 29.8 | 1.8 | 16463 | 2417 (15) | 216 (1) |
| 2009 | 126 | 126 (100) | 0 | 20.4 | 1.5 | 13486 | 12659 (94) | 0 |
| 2010 | 128 | 128 (100) | 0 | 21.7 | 1.5 | 14706 | 14083 (96) | 0 |
| 2011 | 167 | 167 (100) | 0 | 41.7 | 2.8 | 15041 | 14474 (96) | 0 |

^1^ Because lack of data, it is assumed that the number of active vessels in 1998 is equal to 1997.
