# Supplementary material for: Human and climatic drivers affect spatial fishing patterns in a multiple-use marine protected area: The Galapagos Marine Reserve
Source: PLoS One. 2020 Jan 23;15(1):e0228094. doi: 10.1371/journal.pone.0228094 (PMC6977758; doi:10.1371/journal.pone.0228094)
Supplement: S2 Table — (DOCX) [file pone.0228094.s002.docx]

**S2 Table. Fishing fleets estimated mean core areas and distribution ranges (in km^2^), according to port interviews and observer onboard data collected in the Galapagos Marine Reserve from 1997 to 2011.**  Source: Participatory Programme of Fisheries Monitoring and Research (PIMPP, in Spanish). SD: Standard deviation.

| **Port** | **Sampling method** | **Core Area** | | **Distribution range** | |
| --- | --- | --- | --- | --- | --- |
|  |  | **Mean** | **SD** | **Mean** | **SD** |
| Puerto Ayora | Port interviews | 5221.2 | ± 5529.9 | 24581.3 | ± 25564.6 |
|  | Observer onboard | 8751.6 | ± 7851.4 | 38911.5 | ± 36070.3 |
| Puerto Villamil | Port interviews | 904.2 | ± 499.5 | 6577.2 | ± 4098.1 |
|  | Observer onboard | 1706.5 | ± 209.0 | 9163.2 | ± 2314.9 |
| Baquerizo Moreno | Port interviews | 4143.1 | ± 4347.4 | 17844.0 | ± 17360.9 |
|  | Observer onboard | 17724.2 | ± 4899.9 | 72419.5 | ± 20181.1 |
