# Supplementary material for: Human and climatic drivers affect spatial fishing patterns in a multiple-use marine protected area: The Galapagos Marine Reserve
Source: PLoS One. 2020 Jan 23;15(1):e0228094. doi: 10.1371/journal.pone.0228094 (PMC6977758; doi:10.1371/journal.pone.0228094)
Supplement: S3 Table — (DOCX) [file pone.0228094.s003.docx]

**S3 Table. Fishing fleets estimated site fidelity (IOR95) to similar core areas and distribution ranges, according to port interviews and observer onboard data collected in the Galapagos Marine Reserve from 1997 to 2011.** Source: Participatory Programme of Fisheries Monitoring and Research (PIMPP, in Spanish). SD: Standard deviation.

| **Port** | **Sampling method** | **Core Area** | | **Distribution range** | |
| --- | --- | --- | --- | --- | --- |
|  |  | **IOR95** | **SD** | **IOR95** | **SD** |
| Puerto Ayora-Baquerizo Moreno | Port interviews | 0.013 | ± 0.021 | 0.090 | ± 0.084 |
|  | Observer onboard | 0.137 | ± 0.083 | 0.250 | ± 0.090 |
| Puerto Ayora-Puerto Villamil | Port interviews | 0.002 | ± 0.004 | 0.050 | ± 0.039 |
|  | Observer onboard | 0.003 | ± 0.006 | 0.043 | ± 0.025 |
| Puerto Villamil-Baquerizo Moreno | Port interviews | 0.000 | ± 0.000 | 0.013 | ± 0.020 |
|  | Observer onboard | 0.000 | ± 0.000 | 0.040 | ± 0.036 |
