# Supplementary material for: Human and climatic drivers affect spatial fishing patterns in a multiple-use marine protected area: The Galapagos Marine Reserve
Source: PLoS One. 2020 Jan 23;15(1):e0228094. doi: 10.1371/journal.pone.0228094 (PMC6977758; doi:10.1371/journal.pone.0228094)
Supplement: Attachment — Submitted filename: Review of PONE_D_19_26580_Castrejon_Charles.pdf [file pone.0228094.s004.pdf]

Review of PONE-D-19-26580. “Human and climatic drivers affect spatial fishing patterns in a multiple-use marine protected area: the Galapagos Marine Reserve”. Castrejon, M. and A. Charles

This study evaluated how the spatiotemporal allocation of fishing effort for lobsters in the Galapagos multiple-use Marine Protected Area was affected by the interaction of diverse climatic and human drivers, before and after implementation of no-take zones. The study used GIS data on fishing effort and BRTs to attempt to identify how these drivers affected spatial fishing patterns. The paper concludes that the boom-and-bust exploitation of the sea cucumber fishery and the global financial crisis (2007-2009), rather than no-take zone implementation, were the most important drivers affecting the distribution of fishing effort for lobsters across the archipelago. The study is spatially and temporally extensive (most of the Galapagos Islands, 1997-2011), the data are fairly well-analyzed and interpreted, and the manuscript is well-written. I have no major disagreements with the conclusions. I also have some sympathy with the suggestions that the MPA network placement could be revisited or even revised, and outside the network TURFS encouraged. My comments are mostly to assist the authors with publication.

Major Comments.

1. This is a paper that, in effect, quantifies spatial and temporal trends in fishing effort of a lobster fishery in a developing country. Yet the emphasis chosen is how this data informs effects of MPA implementation. It is highly commendable that the study includes before and after implementation data. In fact, this is such an important aspect of the study, I would recommend that the authors stress this point more in the paper. However, this MPA network is also well-known as a “classic” case where fishers ensured that no-take zones were NOT placed where fishers fished (Edgar et al 2004 Ref. 22 in this manuscript). That is, it is a case where you might NOT expect much change in spatial effort in the lobster fishery pre- and post-implementation of the MPA network (which is what they found). This very important point is not even mentioned until Lines 999-1010 in the Discussion. I recommend that you mention this much earlier in the paper, probably in the Abstract and Introduction.
2. You place a substantial amount of faith in the “explanatory” powers of your BRTs. This needs to be tempered a fraction. Table 4 indicates that the deviance explained by the BRTs is 29.47% (Regional), 35.73% (PV), 32.66% (PA) and 15.74% (BM). If I understand Figures 7-10 and Table 4 correctly, this amount of deviance explained is then partitioned among 14 potential explanatory predictor variables. Thus, Distance from Port, your strongest driver in the Regional analysis, explains 22.4% of 29.47% (i.e. 6.6%) of the variance. For BM, your strongest driver, Longitude, explains about 17% of 15.74% (i.e. 2.7%). Clearly all of the weaker drivers “explain” very small percentages of the spatial trends. Thus, describing small peaks and troughs in the trends shown in individual panels in Figs. 7-10 is almost describing details

unnecessarily. That said, I agree that the major 6 drivers in the BRTs are as you indicate at Lines 1045-47.

3. The spatial scale at which you measure effort (2.25 km^2) may be rather coarse to be making confident statements about the lack of evidence for “fishing the line”. Many of the studies of spillover (see references cited below at Line 86) often report this effect at much smaller spatial scales than this. You should at least acknowledge this point.
4. The Discussion is far too long and repetitive (17 pages, with a Summary of almost 7 pages). This should be condensed considerably.
5. Lines 93-97 (Introduction) and 952-954 (Discussion) “…to our knowledge, no study has examined yet how fishers respond to those situations in which they have to cope simultaneously with implementation of an MPA, and with the interaction of external drivers…”. A relevant, similar, example is the perceived effect of the rezoning of Australia’s Great Barrier Reef Marine Park in 2004 on local fisheries described by Fletcher WJ et al (2015) Large-scale expansion of no-take closures within the Great Barrier Reef has not enhanced fishery production. Ecol. Appl. 25: 1187-1196 and critiqued by Hughes TP et al (2016) A critique of claims for negative impacts of marine protected areas on fisheries. Ecol. Appl. 26: 637-641. I would recommend that you cite these two papers.

Minor Comments.

Abstract.

Line 30. MP Area (omit s).

Line 31. Note change in font size of text at full stop.

Line 37. Unfeasible (not infeasible).

Introduction

Line 73. “…pay greater attention to the human dimensions of MPAs [10,11]…” In addition references 10 and 11 cited, both by the authors of the current paper, a very relevant example possibly worth citing here would be: Alcala A.C. and G.R. Russ (2006). No-take marine reserves and reef fisheries management in the Philippines: A new people power revolution. Ambio 35(5): 245-254.

Line 86 (and 198). In addition to the Kellner reference (15) on spillover and fishing the line, which is a modelling paper, and Ref. 26 (line 198) and Ref. 70 (line 1264) regarding spillover, three excellent empirical papers on spillover that could be cited are the review by Halpern BS et al (2010) Spillover from marine reserves and the replenishment of fished stocks. Env. Cons. 36: 268-276; Goni R et al (2010) Net contribution of spillover from a marine reserve to fishery catches. Mar. Ecol. Prog. Ser. 400:233-243 (on lobsters in the Mediterranean); and Kerwath SE et al (2013) Marine protected area improves yield without disadvantaging fishers. Nature Communications 4:2347. You should also acknowledge the possibility of larval (as opposed to adult) export from reserves to fished areas, for example: Harrison HB et

al (2012) Larval export from marine reserves and the recruitment benefit for fish and fisheries. Current Biology 22:1023-1028.

Lines 93-97. Note major point 5 above.

Lines 116-117. Indicate here the year when the MPAs were implemented (2000).

Materials and Methods.

Line 198. "….and spillover to fishing grounds may occur ([26]"

Lines 208, 209. Tourist or tourism (not touristic).

Lines 273-274. Why calculate effort by dividing catch by catch-per-unit-effort (CPUE)? Surely you measured catch and effort directly to calculate CPUE?

Line 302. ..affected by the potential drivers (add potential).

Lines 310-312. You make it clear that the re-zoning was confounded by the sea-cucumber over-exploitation phase (see also Table 3). Thus, when you talk of changes to effort associated with the zoning (e.g. Lines: 600-605, 989-990, 1035-1036) you must acknowledge this confounding. At lines 1096-1099 you DO acknowledge the confounding, and should in other places in the manuscript.

Lines 367, 401, 407. Insert "the" before: normality assumption, input field, z score.

Results.

Line 505. (Fig. 2a, b, c) should read (Fig. 2d, e, f).

Line 520. (Fig. 2d, e, f) should read (Fig. 2a, b, c).

Line 601. Acknowledge confounding of zoning and sea-cucumber over-exploitation phase.

Line 644. ..the eastern part, ..the southeastern part (insert the).

Line 678. Fishers (add s).

Line 702. These types of fishers..

Line 718. Suggest (not suggests).

Line 888. Western side of

Discussion.

Lines 952-954. Note comment re Fletcher et al (2015) and Hughes et al (2016) above.

Lines1174-1176. Good point. The lobster recovery may not be related to the implementation of the MPAs.

Lines 1188-1190 and 1218-1220. When suggesting a re-evaluation of the MPA zoning, you must be clear about why the MPAs were established: conservation, fisheries management, or both.

Lines 1194. The TURFS suggestion outside the MPAs is a good one.

Lines 1199-1201. Alcala and Russ (2006) could be cited here also.

Lines 1224-1227. Why would an MPA network placed in a biased manner help the fishery if it was set up to avoid the fishery?

Line 1238. ..replicates.

Line 1241. Thirdly (not Fourthly).

Lines 1249-1262. In addition to the Kay example in the Channel Islands, which is a good one, you could also mention the Goni et al (2010) lobster example from the Mediterranean.

Line 1264. Ref. 70 in support of the idea of spillover is inadequate. See references to cite on spillover suggested above.

Line 1291. To support (not the support).

Fig. 2. What do the dark grey and light grey shaded areas of time represent? El Nino/La Nina? Specify in caption.

Figs. 3 and 4. What are the units here? Effort (diver hours)? Specify in caption.

Figs. 3-6. I find it difficult to differentiate Fig. 3 from 4, or Fig. 5 from Fig. 6, simply by eye.

Figures 7-10. Specify acronyms for all of the predictor variables in the caption of Fig. 7, then refer to this in the captions of Figs. 8-10. Reader must be reminded what these variables are in the caption.

Fig. 7. I agree, NearNTZ has no pattern.

Table 1. Caption. Sampling method (not smapling).

Table 3. Caption Line 2: occurring (not occurred).

Table 4. Perhaps call the variables “Predictor Variables” in the caption?
