# Supplementary material for: Human and climatic drivers affect spatial fishing patterns in a multiple-use marine protected area: The Galapagos Marine Reserve
Source: PLoS One. 2020 Jan 23;15(1):e0228094. doi: 10.1371/journal.pone.0228094 (PMC6977758; doi:10.1371/journal.pone.0228094)
Supplement: Attachment — Submitted filename: Response to Reviewers.pdf [file pone.0228094.s008.pdf]

January 5th, 2020

Heather M. Patterson, Ph.D.
Academic Editor
PlosOne

Dear Dr. Patterson,

Please find enclosed the revised version of the manuscript entitled "*Human and climatic drivers affect spatial fishing patterns in a multiple-use marine protected area: the Galapagos Marine Reserve"* by Mauricio Castrejón (corresponding author) and Anthony Charles.

As required, a revised version of our manuscript that addresses the points raised during the review is submitted as a single Word document file. Given the novel nature of the topic and lack of previous long-term quantitative studies on Galapagos marine zoning, the paper required a longer treatment that the normal article length for PlosOne.

Also please find attached a rebuttal letter that contains our responses to each point raised by the academic editor and reviewers. We are grateful for the insightful and constructive comments made by all of you. We accepted all your suggestions. We have written the discussion in a more focused and concise manner, as requested.

The Galapagos National Park Service is the owner of the data used for this paper. Therefore, there are legal restrictions on sharing a de-identified data set. However, the data underlying the results presented in the study are available on request at investigacion@galapagos.gob.ec. We confirm that other researchers would be able to access the data set in the same manner as we did, and we did not have any special access privileges that others would not have.

We hope you will be pleased with this new version of the manuscript. Please do not hesitate to contact me with any questions or concerns. Thank you very much for your consideration. We look forward to receiving the acknowledgment of the manuscript and your decision.

Sincerely yours,

Dr. Mauricio Castrejón
Interdisciplinary PhD Program
Dalhousie University
Halifax, Canada
mauricio.castrejon@dal.ca

PONE-D-19-26580R1

Human and climatic drivers affect spatial fishing patterns in a multiple-use marine protected area: the Galapagos Marine Reserve

Rebuttal letter

We acknowledge useful comments provided by the academic editor and one referee. All their suggestions have been included in the revised version of the manuscript. In this letter, we explain how and where the corrections have been incorporated into the text. We follow the editorial order and have numbered referees' suggestions and comments.

Editorial comments

The manuscript is much improved, so I thank the authors for their efforts and Reviewer 2 is satisfied that all the suggestions have been address (Reviewer 1 was not available). That said, Reviewer 2 has noted that the Discussion still requires some work and I agree. The authors should emphasis the important findings first, rather than leading with the 'fishing the line' story. I still think the Discussion could be trimmed down to be more concise as well so I encourage the authors to make their revisions with that in mind. I have some minor editorial corrections as well.

That said, I think these changes can be made relatively quickly and that a more focused and concise Discussion will improve the paper. Once these changes have been made the paper can be accepted. I look forward to seeing the final version of this interesting and timely paper in the near future.

> R: We have written the Discussion section in a more focused and concise manner, emphasizing the most important findings first. We shifted the material around and consolidated the discussion into four main subsections. The first of these is about the 'big picture' of change in the Galapagos, in response to the drivers of change analyzed. The second subsection focuses on the detailed assessment of what caused dynamics of fishing effort. The third brings together all the no-take zones analysis. The fourth summarizes the conclusions and lessons learned. The whole Discussion section was edited and reduced.

Minor Comments

Line 30: Rewrite as 'Assessments of the effectiveness of marine protected areas (MPAs)'
R: Suggestion taken.
Line 65: Write as 'particularly those designed for multiple use' (no hyphen in multiple use here'
R: Suggestion taken.
Line 67: I think this should be 'in which as assessment of the performance of MPAs'
R: Suggestion taken.
Line77: Should be 'processes'
R: Suggestion taken.
Line 146: Do not introduce the abbreviation 'ENSO' as it is only used once again in the manuscript. It is PLoS formatting that abbreviations must be used at least 3 times if they are introduced.
R: Suggestion taken.
Line 185: Just abbreviate the scientific name here as *P. penicillatus* as the full name has already been provided
R: Suggestion taken.
Line 194: I would use MPA rather than marine reserve here to be consistent in the terminology.
R: Suggestion taken.

Line 234: Do not introduce the abbreviation ‘GPS’ as it is not used again in the manuscript.
R: Suggestion taken.
Line 309: What is ‘GNP’? This has not been defined yet. I would just spell it out.
R: Suggestion taken.
Line 403: Do not introduce the abbreviation ‘ENSO, just spell it out.
R: Suggestion taken.
Line 1057: Should be ‘shellfish fisheries’
R: Suggestion taken.
