# Supplementary material for: Human and climatic drivers affect spatial fishing patterns in a multiple-use marine protected area: The Galapagos Marine Reserve
Source: PLoS One. 2020 Jan 23;15(1):e0228094. doi: 10.1371/journal.pone.0228094 (PMC6977758; doi:10.1371/journal.pone.0228094)
Supplement: Attachment — Submitted filename: PLoS editorial comment_Spatial fishing patterns in the GMR.docx [file pone.0228094.s005.docx]

**PLoS editorial comment_Spatial fishing patterns in the GMR**

The issue with this manuscript is that it is just far too long and wordy. It would be much more accessible for the reader if it was written in a more concise manner. Instead, it is very repetitive and often includes details that are not germane to the actual study. There are also too many figures and some should be moved to the Supporting Information, too many sub-headings in the methods, results and discussion (every paragraph does not need its own sub-heading) and too many acronyms, that are often not used or are redefined over and over which is unnecessary. Please only introduce an acronym if it is used several times again in the manuscript and then use it consistently; it only has to be defined once.

The Discussion is a particular problem and is 17 pages on its own, which is just far too long and there are a number of paragraphs that add little and should be deleted. The text overall needs work and contains quite a few grammatical errors.

Line 30: Do not capitalise ‘marine protected areas’

Line 31: Looks like the font size at the end of this line changes

Line 35: Write as ‘interpretation of assessments of’

Line 36: delete ‘assessments’

Line 39: ‘long-term, spatially-explicit’

Line 43: delete ‘(GIS)’ here as this is not used again in the abstract

Line 55: Do not capitalise ‘protected areas’

Line 64: ‘spatially-demarcated areas’

Lines 70-71: don’t understand this text offset by en dashes. Doesn’t follow the previous text so either delete or rewrite.

Line 72: Should the ‘and’ before ‘policy’ be deleted? Doesn’t make sense as written

Line 73: replace the hyphen with a comma and delete the comma after ‘countries’

Line 80: delete the comma

Line 94: no study has yet examined’

Line 95: delete the comma after ‘MPA’

Lines 104-105: Not sure what this means ‘potentially misleading….’ How can you mislead something that has been adopted? Please clarify or delete.

Line 110: Delete the comma

Line 11: Delete the comma

Lines 112-113: Move this to the beginning of the sentence ‘The Galapagos Marine Reserve…..’

Line 135: Add ‘the’ before ‘El Nino’

Lines 137: Move the ‘according to Edgar’ out of the main title of the figure. Either put it in the notes or just include it in the text.

Line 141: Write as 25,144 and ‘distributed on’

Line 150: Delete ‘to’ after ‘provides’

Line 153: Write as ‘large, wooden boats’

Line 169: Write as ‘fishing season since 1999’

Line 170: delete ‘since 1999’

Lines 178-232: This is very long and wordy and much of it does not seem directly relevant to the study. I would cut this back to only the information that is applicable to the study.

Line 179: The abbreviation GMR has already been established and does not need to be established here again so just use ‘GMR’

Line 184: The colon should be a comma

Line 184: Do not introduce the abbreviation PMB

Line 185: Do not introduce the abbreviation IMA

Line 189: 1997-98

Line 201: Delete the apostrophe in miles

Line 208: Should be ‘tourist activities’ and would be good to have an example of what that means.

Line 209: tourist activities

Line 216: Need to define CPUE here and define the abbreviation if you are going to use it

Lines 224-232: Most of this is a repeat from the introduction on why the study is being done so delete

Line 238: ‘spatially-explicit’

Line 242: ‘Fisheries-related data’

Line 245: Table 1

Line 258: delete ‘up’

Line 263: 17,723

Line 272: ‘from 2009’

Line 274: Need to define CPUE at line 216, not here, so just use ‘CPUE’

Line 283: delete the comma at the end of the line

Line 290: Too many sub-headings, should delete some

Line 292: I would say ‘The most potentially relevant’ because there is no evidence these are relevant.

Line 296: Again ‘that potentially affected’

Line 307: ‘boom-and-bust’

Line 312: delete ‘the Galapagos’ as this is not necessary

Line 313: delete the comma

Line 317: Again, delete ‘in Galapagos’

Line 343: Don’t need a part 1 and 2 for the sub-heading

Line 346: abbreviation GIS has already been established and does not need to be established here again so just use ‘GIS’

Line 357: Delete the apostrophe

Lines 370-371: Don’t need a new sub-heading

Line 374: Should be ‘is concentrated’

Line 388: ‘fine-scale distribution’

Line 407: Why are there vertical lines around 1.96?

Lines 447-457: Is all this background on BRT required?

Lines 450-451: Do not capitalise ‘general linear models’ or ‘general additive models’ and if the abbreviations are not used again delete them

Line 489: results should be in the past tense so please check the text

Line 507: Long-term variation

Line 524: This is confusing as it is not clear to me how the authors have decided that the GFC contributed to this. Is this just due to the time overlap? Not sure how Fig 2 demonstrates this either.

Line 531: Use the abbreviation GMR

Line 534: Don’t need a part 1 and 2

Line 561: The authors introduced an abbreviation for core area and distribution ranges on line 539 so they should use them throughout the manuscript or do not introduce the abbreviation

Line 564: Should be ‘were shared’

Line 566: Should be ‘showed’

Line 571: Should be ‘trends of the’

Lines 588-589: This is very awkward and the results section is not the place for this. Just write is as a simple statement of the results (not in italics) ‘Based on these results there is a pattern where…..’

Lines 619-620: Same comment as above.

Line 626: ‘boom-and-bust’

Line 630: Replace ‘In consequence’ with ‘Therefore’

Line 634: Don’t need a part 2

Line 686: ‘fishing effort was concentrated’ and delete the apostrophe at the end of the line.

Line 688: boom-and-bust

Line 698: replace the dash with a comma

Line 702: ‘This type of fisher’

Line 721: pearson’s

Line 724: 35.73%, 32.66%

Line 725: Pearson’s

Line 728: delete ‘VI’

Line 731: Use the abbreviation BRT

Line 745: Just use the abbreviation RN

Line 768: Need a comma after ‘islands’

Lines 781-782: This is confusing as written. Notes there was a decline but the revenue goes up to $11,000? Also, write as US$2000 etc. Please change throughout manuscript.

Line 793: delete ‘VI’

Line 811: replace the dash with a comma

Line 819: delete ‘the random number variable’

Line 829: Replace the dashes with commas

Line 845: Delete ‘a value of’

Line 854: delete ‘VI’

Line 860: delete ‘VI’

Line 866: delete ‘VI’

Line 870: Write as US$2500

Line 871: same as above

Line 873: same as above

Line 886: Should be ‘islands’

Line 942: delete the apostrophe and write as ‘fishing fleet core areas’

Line 944: ‘boom-and-bust’

Line 950: Too many sub-headings

Lines 956-962: This text is repetitive and unnecessary so delete and move the next paragraph up to follow the opening sentence of the paragraph.

Lie 970: ‘boom-and-bust’

Line 981: GMR

Line 985: ‘fishing fleet core areas’

Line 994: Don’t redefine the abbreviation nearNTZ, just use it.

Line 1000: GMR

Line 1008: Move ‘historically’ to line 1009 after ‘areas’

Line 1045: ‘these adaptive responses’? what responses?

Line 1048: ‘although this was not the case for Puerto Ayora’

Line 1053: delete ‘on the water’

Line 1065: Reference not formatted correctly, needs a reference number

Line 1066: Do not capitalise ‘discrete choice models’ and delete the acronym as it is not used again.

Line 1074: Replace the dash with a comma

Line 1075: replace the dash with a comma

Line 1082: Delete ‘fishing fleets’

Line 1086: Reference not formatted correctly, needs a reference number

Line 1111: delete the comma and ‘on the water’

Line 1133: CPUE has already been defined and does not need to be defined again here so just use CPUE

Line 1145: Delete ‘has’

Line 1149: delete ‘probably’

Line 1150: add ‘likely’ before ‘irrelevant’

Lines 1153-1165: This is very confusing. I have no idea why the authors are providing a summary half way through the Discussion. This is unnecessary and repetitive so please delete.

Line 1166: Use ‘GMR’

Line 1173: Use ‘GMR’

Lines 1178-1183: This is a sentence, not a paragraph, so please move it up to join the paragraph above.

Line 1178: Delete both commas

Line 1180: Use ‘GMR’

Line 1185: replace the dash with a comma

Line 1187: replace the dash with a comma

Line 1189: Use ‘GMR’

Line 1192: Use ‘GMR’

Line 1197: Use ‘GMR’

Line 1205: Do not introduce the abbreviation MSC

Lines 1208-1216: Move this up to join the previous paragraph (which is actually a sentence).

Line 1210: Use ‘GMR’

Line 1222: Replace ‘usefulness’ with ‘utility’

Line 1223: EBM has already been defined and does not need to be defined again here so just use EBM

Line 1226: Use ‘GMR’

Line 1231: replace ‘over’ with ‘on’ and do not need to provide the scientific name as this has already been established

Line 1235: Delete ‘Using non-parametric statistics’

Line 1253: Do not introduce the abbreviation SBCI

Line 1254: replace ‘over’ with ‘on’ and abbreviate as P. interruptus

Line 1257: Should be vs.

Line 1259: Don’t need z=0.59

Line 1260: Spell out SBCI

Lines 1263-1270: This is very repetitive and adds nothing to the discussion in my view so I would delete.

Line 1283: Delete ‘enabling’

Line 1290: Delete ‘enabling’

Line 1293: ‘based on the core areas and distribution ranges of fishing fleets’

Line 1303: Delete ‘fishing fleets’

Lines 1305-1315: Again, I find this text unnecessary and it could be deleted.

Lines 1319-1323: This is a sentence, not a paragraph

Line 1321: EBM has already been defined and does not need to be defined again here so just use EBM

Line 1328: ‘should not be taken for granted; MPAs are not a panacea. (delete the rest ‘i.e. a one-size…..’

Line 1332: ‘with global climate change’

Line 1336: delete ‘learned’ and write as ‘is that assessments of MPA effectiveness’
