# Supplementary material for: Human and climatic drivers affect spatial fishing patterns in a multiple-use marine protected area: The Galapagos Marine Reserve
Source: PLoS One. 2020 Jan 23;15(1):e0228094. doi: 10.1371/journal.pone.0228094 (PMC6977758; doi:10.1371/journal.pone.0228094)
Supplement: Attachment — Submitted filename: PLoS editorial comment_GMR.docx [file pone.0228094.s007.docx]

PLoS editorial comment_GMR

Line 30: Rewrite as ‘Assessments of the effectiveness of marine protected areas (MPAs)’

Line 65: Write as ‘particularly those designed for multiple use’ (no hyphen in multiple use here’

Line 67: I think this should be ‘in which as assessment of the performance of MPAs’

Line77: Should be ‘processes’

Line 146: Do not introduce the abbreviation ‘ENSO’ as it is only used once again in the manuscript. It is PLoS formatting that abbreviations must be used at least 3 times if they are introduced.

Line 185: Just abbreviate the scientific name here as P. penicillatus as the full name has already been provided

Line 194: I would use MPA rather than marine reserve here to be consistent in the terminology.

Line 234: Do not introduce the abbreviation ‘GPS’ as it is not used again in the manuscript.

Line 309: What is ‘GNP’? This has not been defined yet. I would just spell it out.

Line 403: Do not introduce the abbreviation ‘ENSO, just spell it out.

Line 1057: Should be ‘shellfish fisheries’
